# Hormone Receptor and ERBB2 Status in Gene Expression Profiles of Human Breast Tumor Samples

**DOI:** 10.1371/journal.pone.0026023

**Published:** 2011-10-13

**Authors:** Anna Dvorkin-Gheva, John A. Hassell

**Affiliations:** Centre for Functional Genomics, Department of Biochemistry and Biomedical Sciences, McMaster University, Hamilton, Ontario, Canada; Health Canada, Canada

## Abstract

The occurrence of large publically available repositories of human breast tumor gene expression profiles provides an important resource to discover new breast cancer biomarkers and therapeutic targets. For example, knowledge of the expression of the estrogen and progesterone hormone receptors (ER and PR), and that of the ERBB2 in breast tumor samples enables choice of therapies for the breast cancer patients that express these proteins. Identifying new biomarkers and therapeutic agents affecting the activity of signaling pathways regulated by the hormone receptors or ERBB2 might be accelerated by knowledge of their expression levels in large gene expression profiling data sets. Unfortunately, the status of these receptors is not invariably reported in public databases of breast tumor gene expression profiles. Attempts have been made to employ a single probe set to identify ER, PR and ERBB2 status, but the specificity or sensitivity of their prediction is low. We enquired whether estimation of ER, PR and ERBB2 status of profiled tumor samples could be improved by using multiple probe sets representing these three genes and others with related expression.

We used 8 independent datasets of human breast tumor samples to define gene expression signatures comprising 24, 51 and 14 genes predictive of ER, PR and ERBB2 status respectively. These signatures, as demonstrated by sensitivity and specificity measures, reliably identified hormone receptor and ERBB2 expression in breast tumors that had been previously determined using protein and DNA based assays.

Our findings demonstrate that gene signatures can be identified which reliably predict the expression status of the estrogen and progesterone hormone receptors and that of ERBB2 in publically available gene expression profiles of breast tumor samples. Using these signatures to query transcript profiles of breast tumor specimens may enable discovery of new biomarkers and therapeutic targets for particular subtypes of breast cancer.

## Introduction

The accurate assessment of the expression of the estrogen and progesterone hormone receptors (ER and PR) and that of ERBB2 is essential to select the appropriate therapy for breast cancer patients [1], [2], [3], [4], [5]. Knowledge of the expression of the latter biomarkers is also advantageous to develop new therapies that may target specific subtypes of breast cancer [6], [7]. ER and PR status is routinely defined by immunohistochemistry (IHC), whereas that of ERBB2 is determined by either IHC or by fluorescence in-situ hybridization (FISH) [8], [9]. However, despite standardization of the methods used to define the status of the hormone receptors and ERBB2 in clinical laboratories, there is a level of subjectivity in these measurements, leading to variability among results obtained by different pathologists and laboratories [10], [11], [12], [13]. It has been suggested that more accurate and less subjective methods would improve the classification of human breast tumors [14].

Global gene expression profiling is widely used to examine the expression of thousands of genes in biological samples [15]. Indeed, this technology has been used extensively in numerous breast cancer studies to: examine the effects of various therapies on gene transcripts [16], [17]; identify differences in gene expression among different tumor tissues [18], [19], [20], [21]; molecularly classify tumors [22], [23], [24]; and to predict prognosis [25], [26], [27] and treatment outcomes [28], [29], [30]. Attempts to use gene expression profiles to identify the ER, PR and ERBB2 status of human breast tumors have also been reported [14], [31], [32]. A single probe set representative of each gene was informative to establish ER, PR and ERBB2 expression in breast tumor samples. However, we wondered whether the specificity and/or sensitivity of this method could be improved by using probe sets representative of multiple genes (gene signatures) whose expression correlated with that of the hormone receptors and ERBB2.

Many peer-reviewed journals require authors to deposit microarray data in public depositories, such as the Gene Expression Omnibus [33] or ArrayExpress [34], thereby making them publicly available for various applications [35]. However, clinical information such as hormone receptor or ERBB2 status of breast tumor samples is not invariably provided with their global gene expression profiles. Knowledge of hormone receptor and ERBB2 status as well as the global gene expression profiles of breast tumor samples may permit more accurate prognostic tests to be developed and would strengthen the value of the many breast tumor gene expression profiles in public depositories.

Here we used 8 independent datasets containing human breast tumor samples profiled on Affymetrix GeneChips to define gene expression signatures predictive of their ER and PR status as well as that of ERBB2. These gene signatures reliably predicted the status of the hormone receptors and that of ERBB2 as assessed by protein (IHC) or DNA (FISH) based tests. Because the largest predictive signature defined in our study comprises only 51 genes, a qRT-PCR based format may be developed that could provide an objective and relatively high-throughput alternative for the IHC-based definitions of hormone receptor and ERBB2 status in patient samples.

## Results

### ER status

**Figure 1:**
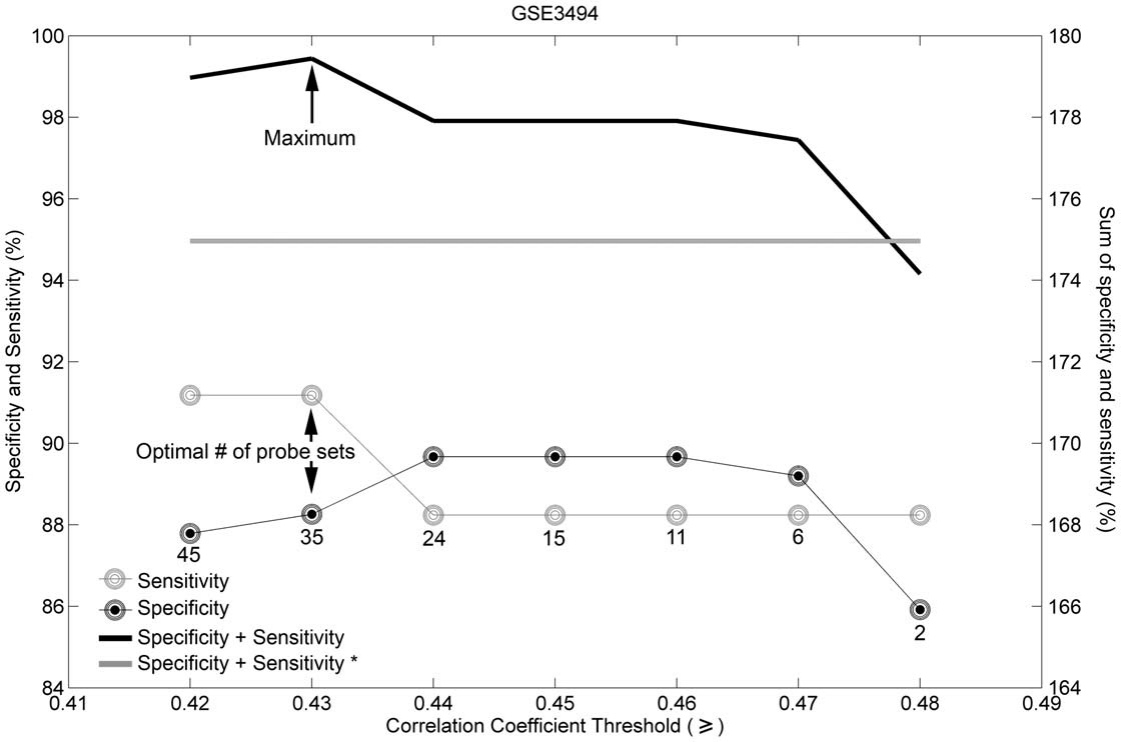
Selecting gene signature predictive of ER status based on sensitivity and specificity. The cutoff is based on Spearman rank correlation coefficients. The number of probe sets in each signature is marked by the number under the lowest curve. Black filled circles – specificity; gray circles – sensitivity; black line – sum of specificity and sensitivity. The optimal number of probe sets was 35, with Spearman rank correlation coefficient cutoff set at 0.43. Gray line and “*” indicate the sum of specificity and sensitivity of the prediction obtained by using a single “best probe set” (“205225_at”).

Figure 1 shows the specificity and sensitivity values for sets of genes predictive of ER status selected by using Spearman rank correlation cutoffs between 0.42 and 0.48. To find the most predictive set of genes, we selected those that yielded the highest combination (here the sum) of specificity and sensitivity values. The identified gene signature consisted of 35 probe sets, representing 24 annotated genes (Table 1). Of these 24 genes, one is the *ESR1* itself, whereas 11 are related to the expression of the ER: the latter include genes (*GATA3, GFRA1*, *IL6ST*, and *STC2*) whose expression correlates positively with that of the ER [36], [37], [38]; genes (*CA12*, *CYP2B6*, *GREB1*, *LIV1*, *TFF1*, and *KDM4B*) whose expression is positively regulated by the ER [37], [39], [40], [41], [42], [43]; and a gene located in close proximity to *ESR1* (*C6orf97*) [44], and whose expression is therefore positively correlated with that of the ER. Importantly, several of these genes are represented by multiple probe sets indicating that they robustly detect their cognate transcripts in breast tumor RNA samples (Table 1). Twelve remaining genes (*ADCY9*, *ANXA9*, *AMFR*, *CELSR1*, *CYP2B7P1*, *FAM176B*, *GAMT*, *KCNK15*, *SCCPDH*, *SCUBE2*, *SSH3*, and *TBC1D9*) have not been previously associated with ER status. Interestingly, *SCUBE2* is reported to positively correlate with PR status [45]. Because our ER signature comprises 24 genes and one probe set for an unknown gene, we refer to the signature as the “24-gene ER signature”. The 24-gene ER signature separated ER-positive tumors from ER-negative tumors with an accuracy of 88.66%, sensitivity of 91.18%, specificity of 88.26%, PPV (Positive Predictive Value) of 98.43% and NPV (Negative Predictive Value) of 55.36% in the 247 training samples (Table 2; p<2.2·10^−16^, Fisher's exact test). To determine whether the predictive performance of a single probe set is sufficient to determine ER status of a sample we used “205225_at”, the probe set with the highest Spearman rank correlation in the 24-gene ER signature (Spearman rank correlation is 0.50; see Table S1), which we termed “best probe set” for the ER predictive signature. It is of interest, that the “best probe set” was the same probe set conventionally used to determine ER status (205225_at; see Table S1). The prediction accuracy of the “best probe set” was 89.07%, sensitivity 89.67%, specificity 85.29%, PPV 97.45% and NPV 56.86% (Table 2; p<2.2·10^−16^, Fisher's exact test). Both the sensitivity and specificity of prediction by using the “best probe set” were lower than were the sensitivity and the specificity of the prediction using the 24-gene ER signature, indicating that the predictive performance of the single “best probe set” is not as high as the performance of our signature.

**Table 1:** Gene signature predictive of ER status.

Gene Symbol	Correlation Coefficient	Gene Title
ADCY9	0.44	adenylatecyclase 9
AMFR	0.44	autocrine motility factor receptor
ANXA9	0.43	annexin A9
	0.45	
C6orf97	0.45	chromosome 6 open reading frame 97
CA12	0.48	carbonic anhydrase XII
	0.48	
	0.47	
	0.47	
	0.47	
CELSR1	0.43	cadherin, EGF LAG seven-pass G-type receptor 1 (flamingo homolog, Drosophila)
CYP2B6 ///	0.45	cytochrome P450, family 2, subfamily B, polypeptides 6, 7
CYP2B7P1		
ESR1	0.50	estrogen receptor 1
FAM176B	0.46	family with sequence similarity 176, member B
	0.43	
GAMT	0.45	guanidinoacetate N-methyltransferase
GATA3	0.45	GATA binding protein 3
	0.48	
	0.47	
GFRA1	0.45	GDNF family receptor alpha 1
GREB1	0.46	growth regulation by estrogen in breast cancer 1
IL6ST	0.44	interleukin 6 signal transducer (gp130, oncostatin M receptor)
	0.44	
KCNK15	0.44	potassium channel, subfamily K, member 15
KDM4B	0.45	lysine (K)-specific demethylase 4B
	0.46	
	0.43	
SCCPDH	0.43	saccharopine dehydrogenase (putative)
SCUBE2	0.46	signal peptide, CUB domain, EGF-like 2
LIV1 (SLC39A6)	0.46	solute carrier family 39 (zinc transporter), member 6
SSH3	0.44	slingshot homolog 3 (Drosophila)
STC2	0.44	stanniocalcin 2
TBC1D9	0.43	TBC1 domain family, member 9 (with GRAM domain)
TFF1	0.44	trefoil factor 1
Unknown	0.45	Not annotated

Each row in the coefficient column represents a probe set. Genes, whose levels of expression were previously reported to correlate with ER status are marked in bold. The rows were sorted alphabetically according to the Gene Symbol. For detailed information on the probe sets see Table S1.

**Table 2:** Correlation of microarray–based expression profiling data with routinely established ER status.

		Total	ER status defined by predictor	Clinical ER status		
				Negative	Positive	p-value*
Training	GSE3494	247	Negative	31	25	<2.2·10–16
			Positive	3	188	
	GSE3494**	247	Negative	29	22	<2.2·10–16
			Positive	5	191	
Validation	GSE2034	286	Negative	68	26	<2.2·10–16
			Positive	9	183	
	GSE7390	198	Negative	52	10	<2.2·10–16
			Positive	12	124	
	GSE2603	97	Negative	41	2	<2.2·10–16
			Positive	0	54	
	GSE20271	144	Negative	54	16	4.227·10–13
			Positive	13	61	
	GSE20194	278	Negative	103	17	<2.2·10–16
			Positive	11	147	

*Fisher's exact test.

**The analysis was performed by using the “best probe set” (“205225_at”). The rest of analyses were performed by using the 24-gene ER signature.

We subsequently tested the predictive performance of the 24-gene signature in 5 independent validation datasets (Table 2). The first validation set (GSE2034) comprised 286 samples; the prediction accuracy was 87.76%, sensitivity 87.56%, specificity 88.31%, PPV 95.31% and NPV 72.34% (Table 2; p<2.2·10^−16^, Fisher's exact test). The second validation set (GSE7390) comprised 198 samples; the prediction accuracy was 88.89%, sensitivity 92.54%, specificity 81.25%, PPV 91.18% and NPV 83.87% (Table 2; p<2.2·10^−16^, Fisher's exact test). The third validation set (GSE2603) is composed of 97 samples; the prediction accuracy was 97.94%, sensitivity 96.43%, specificity 100%, PPV 100% and NPV 95.35% (Table 2; p<2.2·10^−16^, Fisher's exact test). The fourth validation set (GSE20271) contained 144 samples; the prediction accuracy was 79.86%, sensitivity 79.22%, specificity 80.60%, PPV 82.43% and NPV 77.14% (Table 2; p = 4.227·10^−13^, Fisher's exact test). The final validation dataset (GSE20194) comprised 278 samples; the prediction accuracy was 89.93%, sensitivity 89.63%, specificity 90.35%, PPV 93.04% and NPV 85.83% (Table 2; p<2.2·10^−16^, Fisher's exact test).

**Figure 2:**
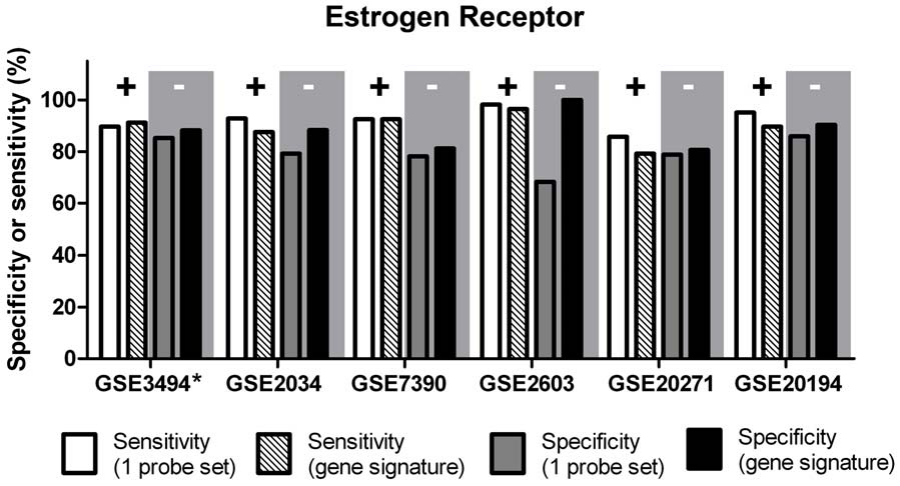
ER status determination: sensitivity (‘+’) and specificity (‘-’) obtained with two different microarray-based methods. The improved feature is highlighted by gray background. * Training set.

Figure 2 and Table S4 depict the sensitivity and specificity levels obtained for the training and the validation sets using the 24-gene ER signature, compared to those derived by using the conventional method of employing a single probe set (205225_at). The sensitivity levels obtained by using a single probe set were relatively high, ranging between 85.71% (GSE20271) and 98.21% (GSE2603); however, the specificity levels were significantly lower than these obtained using the 24-gene ER signature, ranging between 68.29% (GSE2603) and 85.96% (GSE20194; p<0.05, t-test). Hence the 24-gene ER signature significantly improved the specificity levels of ER status prediction (p<0.05, t-test) to range between 80.6% and 100% without adversely affecting sensitivity levels.

### ERBB2 status

**Figure 3:**
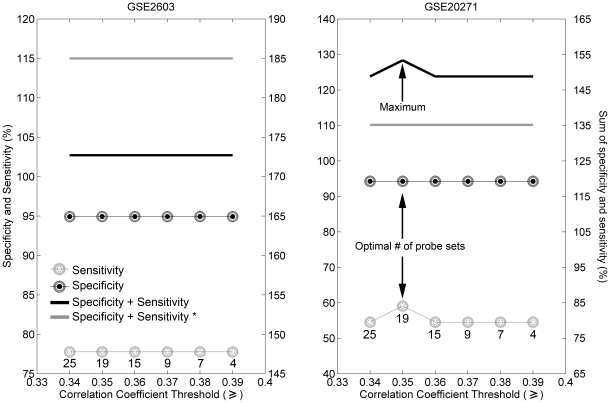
Selecting set of genes predictive of ERBB2 status based on sensitivity and specificity. Cutoff is based on Spearman rank correlation coefficients. The number of probe sets in each signature is marked by the number under the lowest curve. Black filled circles – specificity; gray circles – sensitivity; black line – sum of specificity and sensitivity. The optimal number of probe sets was 19, with Spearman correlation coefficient cutoff set at 0.35. Gray line and “*” indicate the sum of specificity and sensitivity of the prediction obtained by using a single “best probe set” (“203497_at”).

Figure 3 shows the specificity and sensitivity values for gene sets predictive of ERBB2 status selected by using Spearman rank correlation cutoffs between 0.34 and 0.39. For the first training set (GSE2603; Figure 3, *left panel*), the sum of specificity and sensitivity was constant for the examined range of Spearman rank correlation cutoffs. Therefore, we used an additional set of samples for training (GSE20271; Figure 3, *right panel*), which led to the highest combination of specificity and sensitivity values at a cutoff of 0.35, yielding a gene signature consisting of 14 annotated genes (represented by 18 probe sets) and 1 probe set representing an unknown sequence ( Table 3). The *ERBB2* gene and 5 other genes (*CRK7*, *GRB7*, *PERLD1*, *PPARBP*, and *STARD3*) are part of the 17q12-q21 amplicon and are reported to be co-amplified with the *ERBB2* locus [46]. Several of these genes are represented by a number of probe sets indicating that they readily detect their cognate transcripts in breast tumor RNA samples (Table 3). The remaining 8 genes comprising the candidate ERBB2 gene signature have not previously been reported to correlate with ERBB2 expression. Because our signature comprises 14 genes and one probe set representing an unannotated gene we henceforth refer to the ERBB2 predictor as the “14-gene ERBB2 signature”. The 14-gene ERBB2 signature separated ERBB2-positive tumors from ERBB2-negative tumors with an accuracy of 93.18%, sensitivity of 77.78%, specificity of 94.94%, PPV of 63.64% and NPV of 97.40% in the 88 training samples of the first training set (GSE2603; Table 4; p = 1.712·10^−6^, Fisher's exact test). The second training set (GSE20271) comprised 144 breast tumor profiles: the prediction accuracy was 88.89%, sensitivity 59.09%, specificity 94.26%, PPV 65.0%% and NPV 92.74% (Table 4; p  = 2.287·10^−8^, Fisher's exact test). To determine whether the predictive performance of a single probe set is sufficient to determine ERBB2 status, we used the “203497_at”, the probe set with the highest Spearman rank correlation in the 14-gene ERBB2 signature (Spearman rank correlation is 0.45; see Table S2), which we termed the “best probe set” for the ERBB2 predictive signature. For the first training set (GSE2603) the predictive accuracy of the “best probe set” was 96.59%, sensitivity 87.5%, specificity 97.5%, PPV 77.78% and NPV 98.73% (Table 4; p<4.4·10^−8^ , Fisher's exact test). For the second training set (GSE20271) the predictive accuracy of the “best probe set” was 86.11%, sensitivity 40.91%, specificity 94.26%, PPV 56.25% and NPV 89.84% (Table 4; p<5.2·10^−5^, Fisher's exact test). Although predictions by using “best probe set” in both training sets provided similar results, the sensitivity of prediction by using the “best probe set” in the second training set (GSE20271) was very low, reaching 40.91%. Therefore, we suggest that the predictive performance of the 14-gene ERBB2 signature is better than that of the single “best probe set”.

**Table 3:** Gene signature predictive of ERBB2 status.

Gene Symbol	Correlation Coefficient	Gene description
Positive Spearman correlation		
CRK7 (CDK12)	0.38	cyclin-dependent kinase 12
ERBB2	0.42	v-erb-b2 erythroblastic leukemia viral oncogene homolog 2, neuro/glioblastoma derived oncogene homolog (avian)
	0.39	
F2RL1	0.35	coagulation factor II (thrombin) receptor-like 1
GRB7	0.43	growth factor receptor-bound protein 7
IDI1	0.37	isopentenyl-diphosphate delta isomerase 1
ITGB6	0.36	integrin, beta 6
	0.35	
PERLD1	0.37	post-GPI attachment to proteins 3
	0.38	
PPARBP	0.45	mediator complex subunit 1
	0.39	
SEC63	0.37	SEC63 homolog (S. cerevisiae)
STARD3	0.37	StAR-related lipid transfer (START) domain containing 3
TRIM26	0.36	tripartite motif-containing 26
Negative Spearman correlation		
DIRAS2	-0.36	DIRAS family, GTP-binding RAS-like 2
DUSP24	-0.36	serine/threonine/tyrosine interacting-like 1
UBTF	-0.36	upstream binding transcription factor, RNA polymerase I
Unknown	-0.37	Not annotated

Each row in the correlation coefficient column represents a probe set. Genes, within the borders of the ERBB2 amplicon are marked in bold. The list of genes is divided into genes with positive and negative (-) correlation coefficients. For detailed information on the probe sets see Table S2.

**Table 4:** Correlation of microarray–based expression profiling data with routinely established ERBB2 status.

		Total	ERBB2 status defined by predictor	Clinical ERBB2 status		
				Negative	Positive	p-value*
Training	GSE2603	88	Negative	75	2	1.712·10^−6^
			Positive	4	7	
	GSE2603**	88	Negative	78	1	<4.4·10^−8^
			Positive	2	7	
	GSE20271	144	Negative	115	9	2.287·10^−8^
			Positive	7	13	
	GSE20271**	144	Negative	115	13	<5.2·10^−5^
			Positive	7	9	
Validation	GSE20194	278	Negative	218	14	<2.2·10^−16^
			Positive	1	45	
	GSE16446	93	Negative	61	5	<2.2·10^−16^
			Positive	1	26	

*Fisher's exact test.

**The analysis was performed by using the “best probe set” (“203497_at”). The rest of analyses were performed by using the 14-gene ERBB2 signature.

We tested the predictive performance of the 14-gene signature in 2 validation sets (Table 4). The first validation set (GSE20194) is composed of 278 breast tumor profiles; the prediction accuracy was 94.60%, sensitivity 76.27%, specificity 99.54%, PPV 97.83% and NPV 93.97% (Table 4; p<2.2·10^−16^, Fisher's exact test). For the second validation set (GSE16446; 93 breast tumor profiles), the prediction accuracy was 93.55%, sensitivity 83.07%, specificity 98.39%, PPV 96.30% and NPV 92.42% (Table 4; p<2.2·10^−16^, Fisher's exact test). Importantly, the second validation set was obtained from transcript profiles performed on a different type of GeneChip – HG-U133 Plus 2.0. We performed this last validation on data collected from HG-U133 Plus 2.0 GeneChips to determine whether the candidate 14-gene ERBB2 signature was capable of separating ERBB2-positive tumors from their ERBB2-negative counterparts independent of the nature of the Affymetrix arrays to which the transcripts were hybridized.

**Figure 4:**
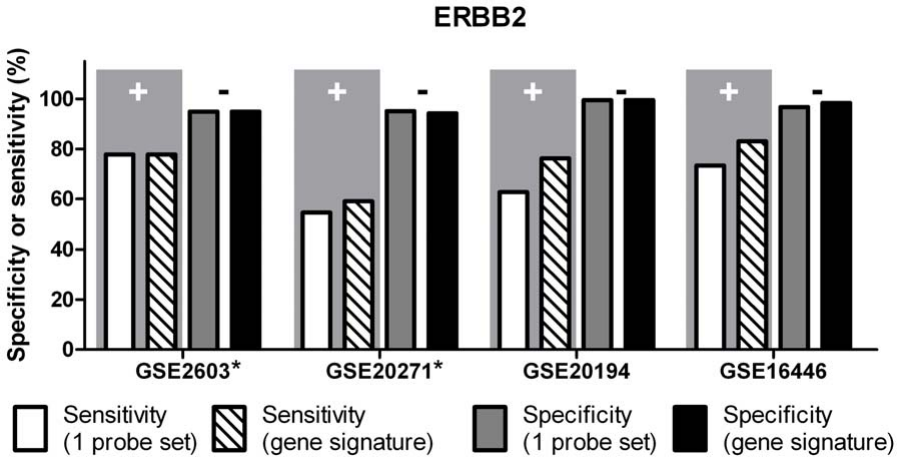
ERBB2 status determination: sensitivity (‘+’) and specificity (‘-’) obtained with two different microarray-based methods. The improved feature is highlighted by gray background. Datasets GSE2603, GSE20271 and GSE20194 were profiled on HG-U133A GeneChips; GSE16446 was profiled on HG-U133 Plus 2.0 GeneChips. * Training set.

Figure 4 and Table S4 depict sensitivity and specificity levels obtained for the training and the validation sets using the 14-gene ERBB2 signature or using the method employing a single probe set (216836_s_at). The specificity levels obtained by using one probe set were relatively high, ranging between 94.94% (GSE2603) and 99.54% (GSE20194); however, the sensitivity levels were significantly lower, ranging between 54.55% (GSE20271) and 77.78% (GSE2603). Whereas the specificity levels were approximately within the same range using the 14-gene ERBB2 signature, the sensitivity levels changed to range between 59.09% (GSE20271) and 77.78% (GSE2603). Importantly, the sensitivity (83.07%) and specificity (98.39%) obtained with HG-133 Plus 2 array (GSE16446) lie within the 95% confidence interval for both sensitivity (CI95%: 45.26–96.84) and specificity (CI95%: 89.11 – 103.4) obtained for HG-U133A arrays, for which our 14-gene ERBB2 signature was originally developed.

### PR status

**Figure 5:**
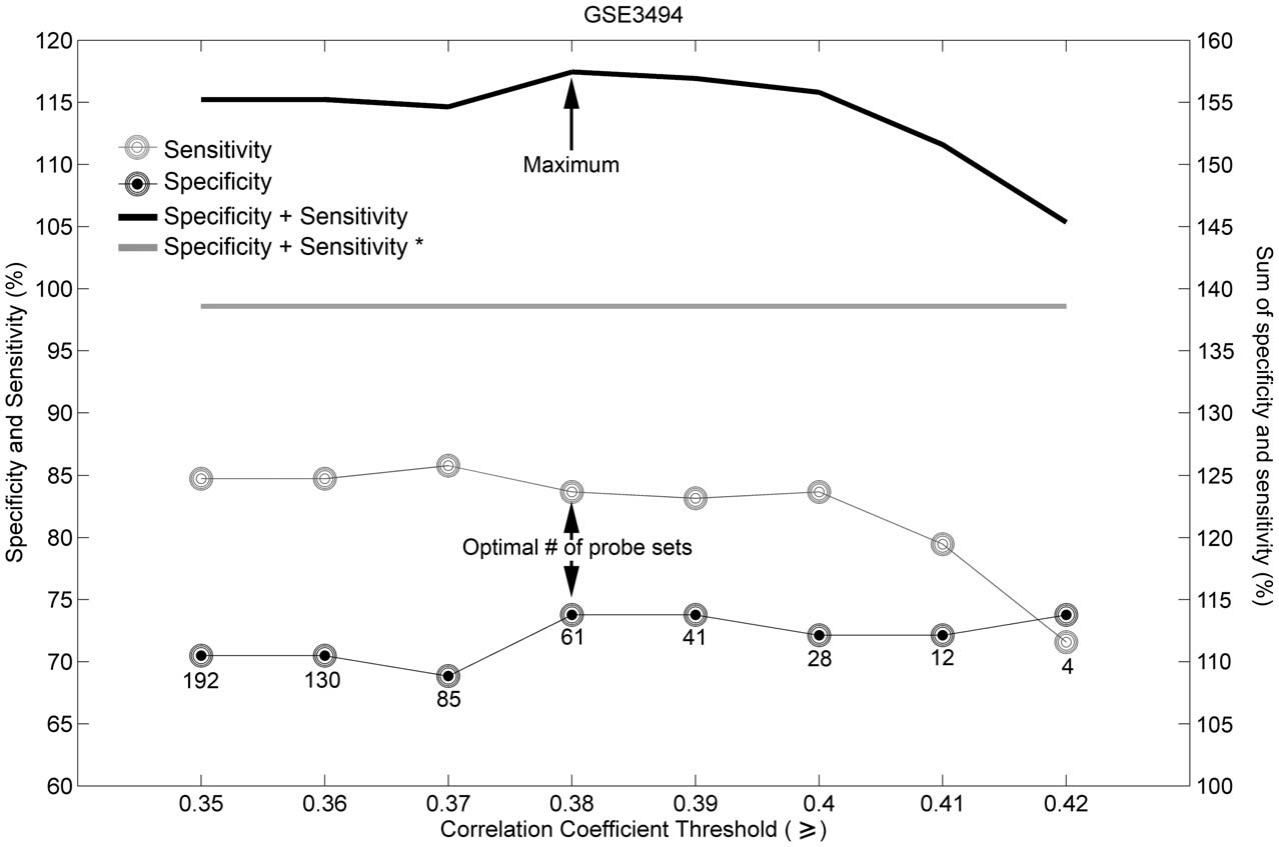
Selecting set of genes predictive of PR status based on sensitivity and specificity. The cutoff is based on Spearman rank correlation coefficients. The number of probe sets in each signature is marked by the number under the lowest curve. Black filled circles – specificity; gray circles – sensitivity; black line – sum of specificity and sensitivity. The optimal number of probe sets is 61, with Spearman correlation coefficient cutoff set at 0.38. Gray line and “*” indicate the sum of specificity and sensitivity of the prediction obtained by using a single “best probe set” (“219197_s_at”).

Figure 5 shows specificity and sensitivity values for genes predictive of PR status selected by using Spearman rank correlation cutoffs between 0.35 and 0.42. The highest combination of specificity and sensitivity values was with a cutoff of 0.38, yielding a gene signature comprising 51 annotated genes (represented by 61 probe sets; Table 5). The PR gene, *PGR*, and 3 other genes (*GATA3*, *STC2* and *GLI3*) [47], [48], [49] are increased in their expression, whereas the expression of 6 genes (*AURKA*, *BUB1*, *CDC20*, *MKI67*, *HJURP*, and *CENPA*) [50], [51], [52], [53], [54] is decreased in PR-positive breast tumors. *GATA3* is expressed in normal mammary epithelial luminal progenitor cells and in the luminal A molecular subtype (ER and/or PR positive tumors) of human breast tumors [24], [55]. Interestingly 11 of the genes comprising the candidate PR gene signature also appeared in the list of genes predictive of ER status (Tables 1 and 5): these genes include *CA12*, *FAM176B*, *GAMT*, *GATA3*, *GFRA1*, *IL6ST*, *KDM4B*, *SCUBE2*, *LIV1*, *STC2* and an un-annotated probe set. The expression levels of *CA12*, *LIV1*, *KDM4B*, *STC2*, *GFRA1*, *ILST6*, and *GATA3* are reported to positively correlate with that of ER [36], [37], [38], [39], [41], [42]. Our results show that all of these 11 genes appear to be up-regulated in both ER-positive and PR-positive samples (see correlation coefficients in Tables 1 and 5). Because our signature comprised 51 genes and one probe set for an unannotated gene we refer to this signature as a “51-gene PR signature”. The candidate 51-gene PR signature contained 2 genes (*HPN* and *MAPT*) whose expression was reported to correlate positively with ER expression [37], [56]; however, these genes did not appear in the ER-predictive signature. The expression of 41 genes out of the 51 annotated genes constituting the PR-predictive signature has not been previously associated with PR status.

The candidate 51-gene PR signature separated PR-positive tumors from PR-negative tumors with an accuracy of 81.27%, sensitivity of 83.68%, specificity of 73.77%, PPV of 90.86% and NPV of 59.21% in the 251 training samples (Table 6; p = 2.3·10^−16^, Fisher's exact test). To determine whether the predictive performance of a single probe set is sufficient to determine PR status we used “219197_s_at”, the probe set with the highest Spearman rank correlation in the 51-gene PR signature (Spearman rank correlation is 0.44; see Table S3), which we termed “best probe set” for PR predictive signature. The prediction accuracy of the “best probe set” was 80.48%, sensitivity 91.05%, specificity 47.54%, PPV 84.39% and NPV 63.04% (Table 6; p<3.3·10^−10^, Fisher's exact test). Although the sensitivity of the prediction by using the “best probe set” was higher than the sensitivity of the prediction by using the 51-gene PR signature, the specificity was very low, reaching only 47.54%. Also the prediction accuracy and PPV were lower when using only the “best probe set”. These findings indicated that the predictive performance of the single “best probe set” is not as high as the performance of the signature.

**Table 5:** Gene signature predictive of PR status.

Gene Symbol	Correlation Coefficient	Gene Title
Positive Spearman correlation		
BBS1	0.40	Bardet-Biedl syndrome 1
	0.40	
BCAM	0.40	basal cell adhesion molecule (Lutheran blood group)
CA12*	0.39	carbonic anhydrase XII
	0.40	
CASC1	0.39	cancer susceptibility candidate 1
*FAM176B*	0.41	family with sequence similarity 176, member B
	0.44	
*GAMT*	0.40	guanidinoacetate N-methyltransferase
GATA3*	0.39	GATA binding protein 3
	0.41	
GFRA1*	0.39	GDNF family receptor alpha 1
GLI3	0.39	GLI family zinc finger 3
HPN	0.39	hepsin
IL6ST*	0.40	interleukin 6 signal transducer (gp130, oncostatin M receptor)
	0.41	
KDM4B*	0.41	lysine (K)-specific demethylase 4B
	0.42	
LAMB2	0.40	laminin, beta 2 (laminin S)
LRRC17	0.39	leucine rich repeat containing 17
LZTFL1	0.39	leucine zipper transcription factor-like 1
MAGED2	0.39	melanoma antigen family D, 2
MAPT	0.39	microtubule-associated protein tau
	0.40	
PDE4A	0.38	phosphodiesterase 4A, cAMP-specific (phosphodiesterase E2 dunce homolog, Drosophila)
PGR	0.41	progesterone receptor
*SCUBE2*	0.44	signal peptide, CUB domain, EGF-like 2
LIV1 (SLC39A6)*	0.38	solute carrier family 39 (zinc transporter), member 6
STARD13	0.39	StAR-related lipid transfer (START) domain containing 13
STC2*	0.38	stanniocalcin 2
WDR19	0.40	WD repeat domain 19
*Unknown*	0.40	Not annotated
Negative Spearman correlation		
AURKA	−0.40	aurora kinase A
	−0.38	
BUB1	−0.41	budding uninhibited by benzimidazoles 1 homolog (yeast)
C16orf61	−0.38	chromosome 16 open reading frame 61
CCNA2	−0.40	cyclin A2
CDC20	−0.40	cell division cycle 20 homolog (S. cerevisiae)
CDCA8	−0.39	cell division cycle associated 8
CENPA	−0.38	centromere protein A
CENPN	−0.38	centromere protein N
CEP55	−0.39	centrosomal protein 55 kDa
DBF4	−0.44	DBF4 homolog (S. cerevisiae)
DDX39	−0.39	DEAD (Asp-Glu-Ala-Asp) box polypeptide 39
DLGAP5	−0.39	discs, large (Drosophila) homolog-associated protein 5
GATAD2A	−0.41	GATA zinc finger domain containing 2A
GTSE1	−0.38	G-2 and S-phase expressed 1
HJURP	−0.39	Holliday junction recognition protein
KIF2C	−0.41	kinesin family member 2C
	−0.41	
KPNA2	−0.41	karyopherin alpha 2 (RAG cohort 1, importin alpha 1)
LAD1	−0.41	ladinin 1
LPIN1	−0.40	lipin 1
MCAM	−0.39	melanoma cell adhesion molecule
MELK	−0.40	maternal embryonic leucine zipper kinase
MKI67	−0.39	antigen identified by monoclonal antibody Ki-67
OR7E37P	−0.39	olfactory receptor, family 7, subfamily E, member 37 pseudogene
PSME4	−0.42	proteasome (prosome, macropain) activator subunit 4
PTTG1	−0.40	pituitary tumor-transforming 1
SLC7A5	−0.40	solute carrier family 7 (cationic amino acid transporter, y+system), member 5
TTK	−0.40	TTK protein kinase

Each row in the correlation coefficient column represents a probe set. Genes, whose levels of expression are reported to correlate with PR are marked in bold. Genes that occur in the signature predictive of ER status are marked in italics. Those genes whose levels of expression have been reported in literature to correlate with ER status are marked by an asterisk. The list of genes is divided into those with positive and negative correlation (-) coefficients. For detailed information on the probe sets see Table S3.

**Table 6:** Correlation of microarray–based expression profiling data with routinely established PR status.

		Total	PR status defined by predictor	Clinical PR status		
				Negative	Positive	p-value*
Training	GSE3494	251	Negative	45	31	2.3·10^−16^
			Positive	16	159	
	GSE3494**	251	Negative	29	17	<3.3·10^−10^
			Positive	32	173	
Validation	GSE20271	144	Negative	63	15	6.1·10^−12^
			Positive	16	50	
	GSE20194	278	Negative	107	22	<2.2·10^−16^
			Positive	50	99	
	GSE9195	79	Negative	9	24	0.1484
			Positive	6	40	

*Fisher's exact test.

**The analysis was performed by using the “best probe set” (“219197_s_at”). The rest of analyses were performed by using the 51-gene PR signature.

We tested the predictive performance of the 51-gene PR signature in 3 validation datasets (Table 6). The prediction accuracy was 78.47%, sensitivity 76.92%, specificity 79.75%, PPV 75.76% and NPV 80.77% in 144 samples of the first validation set (GSE20271; Table 6; p = 6.1·10^−12^, Fisher's exact test). The prediction accuracy was 74.1%, sensitivity 81.82%, specificity 68.15%, PPV 66.44% and NPV 82.94% in 278 profiles of the second validation set (GSE20194; Table 6; p<2.2·10^−16^, Fisher's exact test); however, in the third validation set (HG-U133 Plus 2.0 GeneChip array) the prediction accuracy was 62.03%, sensitivity 62.5%, specificity 60.0%, PPV 86.96%, and NPV 27.27% in 79 samples (GSE9195; Table 6; p = 0.1484, Fisher's exact test).

**Figure 6:**
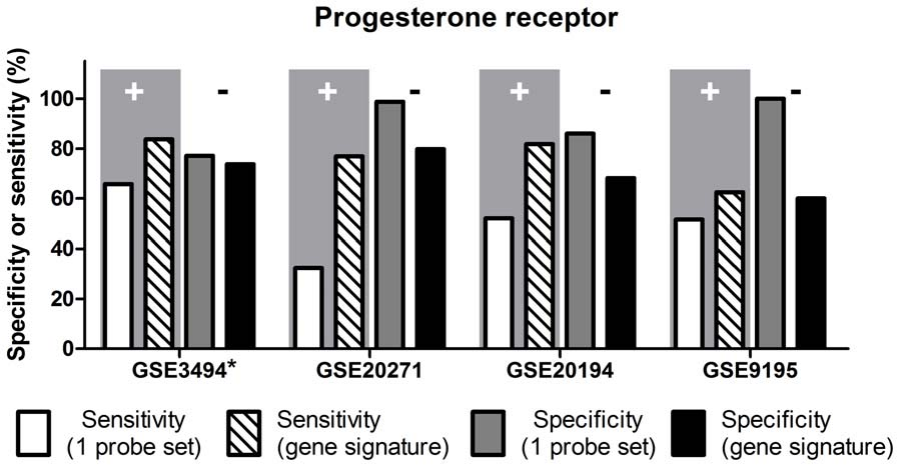
PR status determination: sensitivity (‘+’) and specificity (‘-’) obtained with two different microarray-based methods. The improved feature is highlighted by gray background. Datasets GSE3494, GSE20271 and GSE20194 were profiled on HG-U133A GeneChips; GSE9195 was profiled on HG-U133 Plus 2.0GeneChips. * Training set.

Figure 6 and Table S4 depict sensitivity and specificity levels obtained for the training and the validation sets by using the candidate 51-gene PR gene signature or by using a single probe set (208305_at) to assess PR status in breast tumor specimens. The estimation was performed in the same way as was reported previously to establish PR status based on gene expression profiles. The specificity levels obtained by using a single probe set were relatively high, ranging between 77.05% (GSE3494) and 98.73% (GSE20271); however, the sensitivity levels were lower, ranging between 32.31% (GSE20271) and 65.79% (GSE3494). Whereas using the 51-gene PR signature the specificity levels did not change significantly (p = 0.134, t-test) compared to those using the single probe set, the sensitivity levels were significantly improved (p<0.05, t-test), to range between 76.92% (GSE20271) and 83.68% (GSE3494). The sensitivity (62.5%) obtained with HG-133 Plus 2.0 GeneChip (GSE9195; third validation set) lies within the 95% confidence interval for sensitivity obtained for HG-U133A GeneChip (CI95%: 61.44 – 87.19%). However, the specificity (60.0%) obtained with HG-133 Plus 2.0 GeneChip was lower than the lower limit of the 95% confidence interval for specificity established with the HG-U133A GeneChip (95%CI: 72.08–88.98%). This indicates, that whereas the candidate PR gene signature provides the same level of sensitivity for determining PR status on HG-U133A and HG-U133 Plus 2.0 GeneChips, it provides a lower specificity for determining PR status on HG-U133 Plus 2.0 arrays compared to the HG-U133A arrays for which it was developed.

## Discussion

Global gene expression profiling is widely used in cancer research and the results of these analyses are generally accessible to the scientific community in public repositories. However, these profiles rarely have accessory information concerning the clinically established status of PR, ER or that of ERBB2. Knowledge of the expression of the aforementioned markers could be used to mine publically available gene expression profiles for candidate molecular targets thus aiding efforts to expand the armamentarium of anticancer therapies targeted to these breast tumor subtypes.

Previous studies have demonstrated a correlation between mRNA levels and clinical receptor status as established by IHC, FISH and ligand-binding assays using breast tumor samples [57], [58], [59]. Means have also been established for statistical thresholds for *ESR1, PR* and *ERBB2* transcript levels to assign their expression status in profiled breast tumor samples [14], [31], [32]. These methods use a single probe set to predict ER, PR or ERBB2 status of breast tumor samples. Whereas the latter assays provide good sensitivity for determining ER status and good specificity for those of PR and ERBB2, improvements of these parameters would be desirable to more accurately predict the status of the expression of these genes in breast tumor gene expression profiles.

Our study sought to establish a more accurate specificity for predicting ER status and increased sensitivity for predicting those of PR and ERBB2 while maintaining or improving the sensitivity to predict ER status and to similarly maintain or improve the specificity to predict PR and ERBB2 status. Predictive signatures were developed based on data collected from HG-U133A GeneChips. However, additional GeneChip arrays, HG-U133 Plus 2.0, have been developed (http://media.affymetrix.com/support/technical/datasheets/hgu133arrays_datasheet.pdf), and are increasingly used for global gene expression profiling. Therefore, another goal of our study was to examine the predictive capacity of our signatures using transcript profiles performed on both HG-U133A and HG-133 Plus 2.0 GeneChips to learn whether our predictive signatures perform independently of the nature of the GeneChips used to identify them.

### Gene signature predictive of ER status

The gene signature reported here comprises 24 annotated genes. One of these genes is *ESR1* (estrogen receptor alpha) whereas 11 others have been reported to correlate with the expression of *ESR1* or to be directly regulated by ER [36], [37], [38], [39], [40], [41], [42], [43], [44]. Several of the identified genes are represented by a number of probe sets in the gene signature indicating that these genes have a stable correlation with ER status. Interestingly, one additional gene was found to be reported to positively correlate with PR status [45]. This finding is supported by reports that ER and PR status often correspond with each other [60]. However, this gene was not identified in our PR-predictive gene signature. A plausible explanation for the latter is that we used a high correlation coefficient cutoff to identify the genes belonging to the ER-predictive signature, and hence this gene might have been eliminated during the gene selection process.

Because previously reported methods used a single probe set to determine the hormone and ERBB2 status of tumors, we wished to learn whether a single probe set from the 24-gene ER signature performed as well as the whole signature. To this end we selected the probe set with the highest Spearman rank correlation to the ER status of the sample as the “best probe set”. The best probe set thus identified is identical to that identified in previous studies to determine ER status [14], [31]. The levels of sensitivity and specificity of ER status prediction by using the “best probe set” were lower than the sensitivity of the prediction by using the 24-gene ER signature, indicating that the signature outperformed the “best probe set”.

Previous methods [14], [31] yielded high sensitivity, but a relatively low specificity for predicting ER status (Figure 2).Therefore, we wondered whether we could improve the specificity of ER status prediction by identifying a gene signature to predict ER status. Indeed, our ER-predictive gene signature provides a significantly higher specificity, while maintaining the level of sensitivity. The ER-predictive gene signature we identified was derived by analyzing gene expression data from breast tumor RNA samples profiled on the HG-U133A GeneChip arrays. However, we were unable to find an HG-U133 Plus 2.0 dataset with accompanying clinical information concerning ER status. Future studies will examine the predictive potential of the ER gene signature on HG-U133 Plus 2.0 arrays.

### Gene signature predictive of PR status

The signature predictive of PR status consists of 51 annotated genes, which include the *PGR* (progesterone receptor), and 9 genes (*AURKA*, *BUB1*, *GATA3*, *GLI3*, *STC2*, *CDC20*, *CENPA*, *HJURP* and *MKI67*) that have previously been demonstrated to correlate with *PGR* expression (Table 5; [47], [48], [49], [50], [51], [52], [61]). Interestingly, 11 genes (*STC2*, *GATA3*, *CA12*, *FAM176B*, *GAMT*, *GFRA1*, *IL6ST*, *KDM4B*, *SCUBE2*, *LIV1*, and an ‘unknown’ gene; Tables 1 and 5) out of the 51 genes constituting the PR-predictive signature also appear in our 24-gene ER-predictive signature. These findings are in agreement with other studies reporting that ER and PR status often correlate with each other [60]. Notably, the probe set for the only gene lacking annotation appears in both signatures predictive of PR and ER status indicating a strong connection of the gene reflected by this probe set to ER and PR status. The PR-status predictive signature comprised 2 other genes (*HPN* and *MAPT*) whose expression is positively correlated with ER expression [37], [56]. However, these genes were not identified in our ER-predictive gene signature, probably due to the fact that they had a lower correlation coefficient with ER status than the cutoff established to identify the ER-predictive signature.

The “best probe set” selected from the PR predictive signature was “219197_s_at” (*SCUBE2*). Expression of this gene has not been reported to correlate with PR status of human, however, this gene appears also in our 24-gene ER-predictive signature, and, as has been mentioned earlier, there are studies showing that ER and PR status often show correlation with each other. Specificity of prediction using the “best probe set” was very low, reaching only 47.54% and prediction accuracy and PPV of the were lower than the ones obtained with the 51-gene PR-predictive signature. Therefore, we concluded, that the PR-predictive signature outperformed the single “best probe set”.

Previous method [32] yielded high specificity, but a relatively low sensitivity for predicting PR status (Figure 6). Therefore, we wondered whether we could improve the sensitivity of PR status prediction by identifying a gene signature to predict PR status. By using our gene signature predictive of PR status, we significantly improved the level of sensitivity, while not reducing the level of specificity, as compared to the same measures obtained with 1 probe set (Figure 6).

When tested on data obtained from HG-U133 Plus 2.0 GeneChip arrays, the results differed from the ones obtained from datasets profiled on HG-U133A arrays (Figure 6 and Table 6), indicating, that our candidate PR gene signature needs to be modified to predict PR status of tumor samples profiled on other array types. A plausible explanation for the lower level of performance of the predictive signature on data obtained from HG-U133 Plus 2.0 arrays could be the technical differences in the design of the arrays belonging to HG-U133A and HG-U133 Plus 2.0 types: HG-U133 Plus 2.0 arrays belong to a newer generation of GeneChip arrays, which contain improvements, that result in higher resolution, sharpness, definition and signal uniformity (http://media.affymetrix.com/support/technical/technotes/expression_comparison_technote.pdf). Such technical differences could affect information obtained for the probe sets that were included in our PR signature, among other probe sets.

### Gene signature predictive of ERBB2 status

The ERBB2 predictive gene signature consists of 14 annotated genes, including *ERBB2* and 5 genes (*CRK7*, *GRB7*, *PERLD1*, *PPARBP*, and *STARD3*) located within the *ERBB2* 17q12-q21 amplicon [46]. Several of these genes are represented by multiple probe sets in the ERBB2-predictive gene signature indicating their stable correlation with ERBB2 status.

The “best probe set” selected from the ERBB2 predictive signature was “203497_at”, representing *PPARBP*, a gene, located within the *ERBB2* 17q12-q21 amplicon [46]. The performance of this “best probe set” was tested on two training sets (GSE2603 and GSE20271), that were used to derive the 14-gene ERBB2 signature. The first training set (GSE2603) could not provide us with a clear cutoff for the Spearman rank correlation used to determine the optimal number of genes for the signature (Figure 3). Therefore we needed to test the second training set (GSE20271) as well. The sensitivity of prediction by using the “best probe set” for the second training set (GSE20271) was very low, reaching 40.91%. Therefore, we concluded, that the ERBB2-predictive signature outperformed the single “best probe set”.

A previously described method [14] yielded high specificity levels for predicting ERBB2 status from gene expression profiles using a single probe set (216836_s_at); however, the sensitivity of this method was relatively low. By contrast the specificity levels of our 14-gene signature was unchanged from that reported previously but the sensitivity levels were improved. Additionally, the ERBB2-predictive gene signature also successfully predicted ERBB2 status of gene expression profiles obtained by employing the HG-U133 Plus 2.0 GeneChip (Figure 4 and Table 4).

In summary our findings demonstrate that small gene signatures can be identified in patient breast tumor gene expression profiles that accurately predict ER, PR and ERBB2 status.

## Methods

### Gene expression profiles

As shown in Table 7, to define ER status we used raw CEL files from the following datasets: GSE3494 (247 samples), GSE2034 (286 samples), GSE7390 (198 samples), GSE2603 (97 samples), GSE20271 (144 samples), and GSE20194 (278 samples); to define ERBB2 status we used raw CEL files from GSE2603 (88 samples), GSE20271 (144 samples), GSE20194 (278 samples), and GSE16446 (93 samples); finally to define PR status we used GSE3494 (251 samples), GSE20271 (144 samples), GSE20194 (278 samples), and GSE9195 (79 samples). These aforementioned datasets were downloaded from the Gene Expression Omnibus depository [33]. All samples were profiled on Affymetrix HG-U133A GeneChips (Affymetrix, Santa Clara, CA, USA), with the exception of GSE16446 and of GSE9195, which employed Affymetrix HG-U133 Plus 2.0 GeneChips. All the samples were pre-processed with fRMA [62].

**Table 7:** Sources of the samples and methods used to obtain the clinical information about the samples.

	Total number of profiled samples	ER assessment			PR assessment		ERBB2 assessment
		IHC	EIA*	Other assay	IHC	Biochemical assay	IHC or FISH
**GSE2034**	286	9	277	-	-	-	-
**GSE3494**	251	-	-	247 bioche-mical assay		251	-
**GSE7390**	198	198	-	-	-	-	-
**GSE2603**	121	97 either IHC, EIA or Biochemical assay			-	-	88 IHC
**GSE20271**	144	144	-	-	144	-	144 either IHC or FISH
**GSE20194**	278	278	-	-	278	-	278 either IHC or FISH
**GSE16446**	120	-	-	-	-	-	93 FISH
**GSE9195**	79	-	-	79 Ligand binding			

*enzymatic immunoassay – EIA [64].

### Clinical definition of hormonal receptors status and ERBB2 status

Table 7 shows the sources of the samples and the methods used to obtain the clinical status of the ER and PR and that of ERBB2.

### Filtering repeated samples across datasets

Samples for 2 datasets (GSE20271 and GSE20194) were contributed by the University of Texas M. D. Anderson Cancer Center (MDACC, Houston, TX, USA), and as a result, there were 34 samples that were present in both datasets. For all analyses performed in the present study, these repeated samples were removed from GSE20271, reducing the number of usable samples from 178 to 144.

### Single-probe set estimations

Comparing predictive capacity of our signatures to predictive capacity of single probe sets reported to be used in the literature. For all datasets obtained from HG-U133A GeneChips, the one probe set estimation was performed by using “205225_at” for determining ER status [14], [31] , “216836_s_at” for determining ERBB2 status [14], [31], and “208305_at” for determining PR status [32]. Hormone and ERBB2 status was determined by fitting Gaussian distributions into the distribution of expression values of the examined probe set using Expectation-Maximalization (EM) algorithm [63], similar to the method described by Rody et al [31] and by Lehmann et al [32]. For GSE16446 dataset, which was obtained from HG-U133 Plus 2.0 GeneChips, we used the data on bimodal ERBB2 status supplied with the samples.

Comparing predictive capacity of our signatures to predictive capacity of single probe sets with the highest Spearman rank correlation to the hormone and ERBB2 status (“best probe set”). These comparisons were performed for the training sets used to establish the predictive signatures. The probe set with the highest Spearman rank correlation with ER status was “205225_at” (Spearman rank correlation = 0.50), the same probe set as the one used in literature [14], [31]. We used “203497_at” (Spearman rank correlation = 0.45) for determining ERBB2 status and “219197_s_at” (Spearman rank correlation = 0.44) for determining PR status. Hormone and ERBB2 status was determined in the same way as in the previous single-probe set estimation, by fitting Gaussian distributions using Expectation-Maximalization algorithm.

### Finding gene signatures predictive of ER, PR or ERBB2 clinical status of the tumor samples

**Figure 7:**
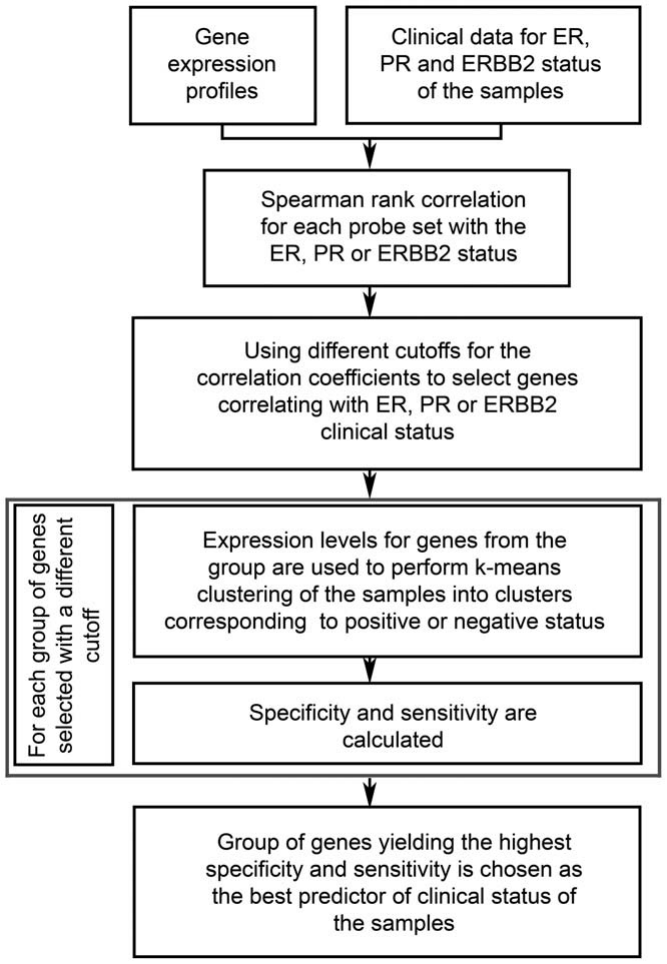
Algorithm for finding the gene signatures predictive of ER, PR or ERBB2 status. The method was used on HG-U133A GeneChip arrays containing 22,283 probe sets. ER – estrogen receptor; PR – progesterone receptor.

Figure 7 describes the algorithm used to find gene signatures predictive of ER, PR or ERBB2 clinical status of the samples. First, global gene expression profiles for the whole training dataset were examined and for each probe set Spearman rank correlation coefficient between its expression levels and clinical status of interest was calculated. The probe sets were sorted by the correlation coefficient, and several groups of genes were selected based on varying correlation cutoff. This way a group of genes selected by using a lower cutoff would contain all the genes belonging to a group selected by using a higher cutoff and additional genes that were filtered out by the higher cutoff. Each group of genes was used for k-means clustering of the samples, in order to define samples with positive and negative status. Then specificity and sensitivity were calculated. Group of genes that led to the highest combination of specificity and sensitivity was defined as a gene signature with optimal predictive ability for the clinical status of interest for the samples in the training set. The same group of genes was used on validation sets, and specificity, sensitivity, accuracy, PPV and NPV were calculated. To derive gene signature predictive of ERBB2 status 2 training sets were needed, since the first set provided constant specificity and sensitivity values for multiple correlation cutoffs.

## Supporting Information

Table S1 **Gene signature predictive of ER status.** Because the gene signature contains only genes with positive Spearman correlation coefficients there is no division based on the coefficients. Genes were sorted alphabetically by their symbol.
(XLS)

Table S2 **Gene signature predictive of ERBB2 status.** The list of genes is divided into those with positive and negative correlation coefficients. Genes were sorted alphabetically by their symbol.
(XLS)

Table S3 **Gene signature predictive of PR status.** The list of genes is divided into genes with positive and negative correlation coefficients. Genes were sorted alphabetically by their symbol.
(XLS)

Table S4 **Hormone and ERBB2 receptors status determination: sensitivity (‘+’) and specificity (‘-’) obtained with two different microarray-based methods.** “One probe set”-by using the single probe set described in the literature. “Signature”-by using our predictive signature.
(XLS)
